# Synthesis and Anti-Tumor Activity of Novel Aminomethylated Derivatives of Isoliquiritigenin

**DOI:** 10.3390/molecules191117715

**Published:** 2014-10-31

**Authors:** Haoran Fu, Yuhang Zhang, Xiqing Wang, Yingzhi Han, Xiao Peng, Thomas Efferth, Yujie Fu

**Affiliations:** 1 School of Chemistry and Environment, Beihang University, Beijing 100191, China; E-Mail: haoran_fu2014@163.com; 2 Key Laboratory of Forest Plant Ecology, Ministry of Education, Northeast Forestry University, Harbin 150040, China; E-Mails: narutohang1991128@163.com (Y.Z.); wangxiqing0622@163.com (X.W.); yingzhi_han@163.com (Y.H.); pengxiao1986@aliyun.com (X.P.); 3 Department of Pharmaceutical Biology, Institute of Pharmacy and Biochemistry, University of Mainz, 55128 Mainz, Germany; E-Mail: efferth@uni-mainz.de

**Keywords:** aminomethylated derivatives of isoliquiritigenin, organic synthesis, Mannich reaction, anti-tumor activity

## Abstract

A series of new aminomethylated derivatives of isoliquiritigenin was synthesized. The structures of the compounds were confirmed by IR, MS, NMR, ^13^C-NMR and elemental analyses. Cytotoxic activities of these derivatives towards the human prostatic cell line PC-3, human mammary cancer cell line MCF-7 and human oophoroma cell line HO-8910 *in vitro* were tested. The IC_50_ values showed cytotoxic activities of some of these new derivatives were relatively strong. Furthermore, tumor growth inhibition *in vivo* of aminomethylated derivatives of isoliquiritigenin **15** was superior to that of isoliquritigenin and reached inhibition rates of 71.68%. The detailed synthesis, spectroscopic data, biological and pharmacologicalactivities of the synthesized compounds were provided.

## 1. Introduction

**Figure 1:**
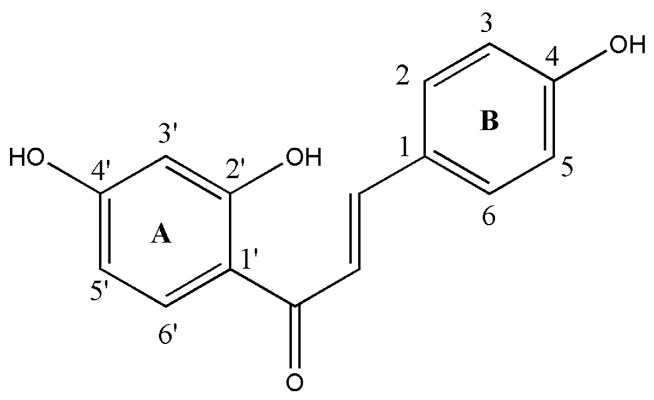
Structure of isoliquiritigenin.

Natural products play a prominent role in oncology because of their potent anticancer activity and good tolerability in normal tissues. Therefore, it is very significant for scientists to screen active compounds from plants and synthesized new derivatives with similar molecular structures as potential anti-tumor drugs or pro-drugs [1,2,3]. As a common active ingredient of many Chinese herbal medicines [4,5], flavonoids reveal little side effects in clinical research [6,7]. Among numerous flavonoids, isoliquiritigenin (ISL) attracted the attention of many scientists. ISL is one of the most important chalcone compound from licorice (Figure 1) and it has a variety of biological activities, such as anti-tumor, anti-virus, anti-free radical, anti-lipid peroxidation and anti-HIV activity, among which the anti-tumor effect came into the focus in recent years [8,9,10,11,12].

The promising anti-tumor effect of ISL stimulates research to modify the chalcone structures, in order to obtain novel derivatives with even stronger anti-tumor activities. So far, ISL and its derivatives have been shown to exert anti-tumor activities by the following mechanism: inhibition of cell proliferation [13,14]; induction of apoptosis [15,16], inhibition of angiogenesis [17], anti-oxidative effects leading to enhanced expression of tumor suppressor genes and decreased expression of oncogenes [18].

In order to enhance water-solubility, bioavailability, and tumor inhibitory activities of ISL, we used ISL as lead compound while remaining its hydroxyl and modifying side chains in A ring of chalcone with nitrogen heterocyclic ring compounds and open-chain amino through Mannich reaction [19,20,21]. We chose the Mannich reaction in our study, because the reaction products Mannich alkaline compounds have a quite wide range of biological activity, such as antibacterial, anticonvulsive, anti-inflammatory, anti-tumor activities [22,23,24,25]. For this reason, Mannich reactions are widely used in medicinal chemistry [26]. To the best of our knowledge, the synthesis of ISL derivatives with different side chains in the A ring containing nitrogen atoms has not been reported yet.

In our present study, chalcone was first synthesized by a hydroxyl-protection method and a consequent condensation reaction was performed in a base environment [27,28]. Then, different nitrogen atoms side chains (heterocyclic azo and open chain amino) were attached to the A ring by Mannich reactions. To test the cytotoxic activities of these derivatives *in vitro*, human prostatic PC-3, human mammary cancer MCF-7 and human oophoroma HO-4980 cells were used. Additionally, the *in vivo* anti-tumor activity of the compound with the lowest IC_50_ value was tested in the mice transplanted with murine S180 sarcoma cells. Our results provide potential ISL derivatives for anti-tumor therapy in the future.

## 2. Results and Discussion

### 2.1. Chemistry

ISL has several active spots, and the two hydroxyl groups in ring A partly attributed to its anti-tumor activity [29]. ISL reveals poor water-solubility. It may be possible to improve its water-solubility by attaching an amino-group to ring A. Meanwhile, by structure-activity-relationship analyses of a series of anti-cancer drugs, we found that many functional groups in drugs contained nitrogen. As a result, heterocyclic azo-groups and open chain amino-groups were chosen to modify ring A by the Mannich reaction. 

**Scheme 1:**
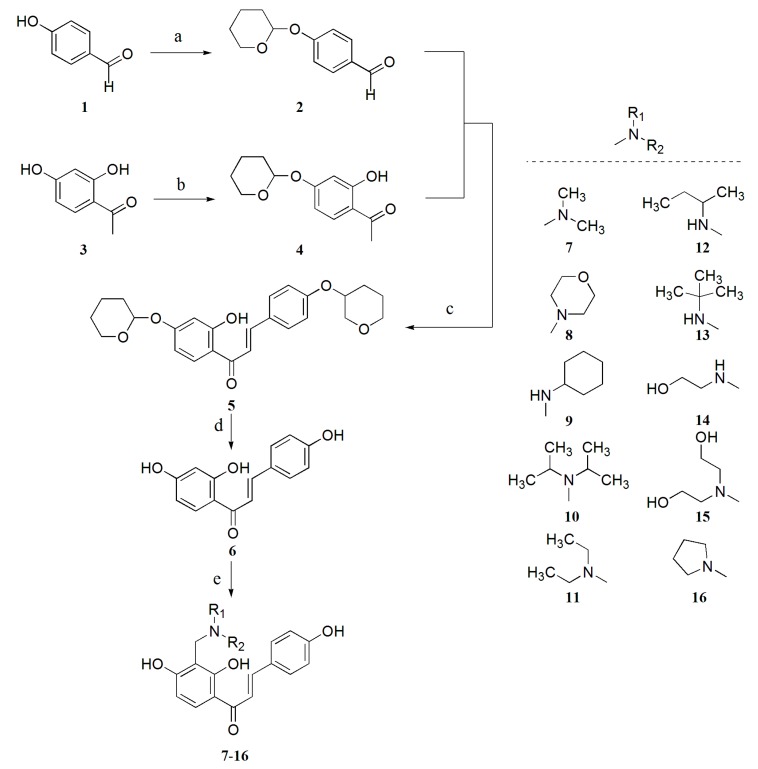
The route of synthetic isoliquiritigenin derivatives.

*Reagents and conditions*: (**a**) pyridine-PPTS, 2,3-dihydropyran, CH_2_Cl_2_, RT, 4 h; (**b**) pyridine-PPTS, 2,3-dihydropyran, NaHCO_3_, CH_2_Cl_2_, RT, 4.5 h; (**c**) Ba(OH)_2_, CH_3_OH_,_ 45 °C, 12 h; (**d**) PTSA, CH_3_OH, RT, 6 h; (**e**) amine, CH_3_CHO, CH_3_COOH, 16–18 °C.

According to this idea, we achieved the synthesis of novel derivatives **7**–**16** (Scheme 1). In our experiment, Substrate **1** (*p*-hydroxybenzaldehyde) and **2** (2,4-dihydroxyacetophenone) were first separately protected by 2,3-dihydropyrane in the presence of pyridine-PPTS, and compounds **3** and **4** were obtained. Then, a condensation reaction of compounds **3** and **4** was conducted with Ba(OH)_2_ as catalyst and CH_3_OH as solvent at 40 °C. Intermediate product **5** was obtained, which was converted to chalcone **6** (ISL) in good yield by deprotection using PTSA. This method using hydroxyl-protection before condensation reaction led to obviously higher yields compared with classical synthesis ways. Next, compounds **7**–**16** were all synthesized by Mannich reactions using chalcone **6**, formaldehyde and amine in one-pot syntheses. 

Interestingly, in the Mannich reaction, extending reaction times or enlarging reactant amounts did not effect the final yields. However, byproducts considerably increased. When Mannich reaction was stopped immediately after adding reagents, the highest yields were achieved. Mannich reactions are often accompanied by many side reactions [30], and with several reaction loci, not only one but several aminomethyl groups attached to the benzene ring of ISL during the reaction process. Besides, we found that the addition of a little bit excessive aldehyde and amine also contributed to higher yields.

### 2.2. Evaluation of Biological Activity

#### 2.2.1. Cytotoxicity Assay

We examined the cytotoxicity of ISL nitrogenous derivatives against three cancer cell lines (PC-3, MCF-7 and HO-8910) and normal human THP-1 cells using MTT growth inhibition assays [31]. ISL was used as a positive control. For THP-1, all derivatives and ISL showed almost no cytotoxicity against normal cells. The IC_50_ values were all above 100 µM. The IC_50_ values for the three cancer cell lines were listed in Table 1. Although not all of these compounds exhibited considerable cytotoxicities against these cell lines, some still showed good cytotoxicities *in vitro* experiment.

**Table 1:** Cytotoxicity of isoliquiritigenin (ISL) derivatives.

Compound	R_1_	R_2_	Cytotoxicity (IC_50_, μM)				mp, °C
			PC-3 ^a^	MCF-7	HO-4980	THP-1	
**7**	Me	Me	>100	>100	>100	>100	>176
**8**	CH_2_CH_2_OCH_2_CH_2_		48.64	>100	96.43	>100	>180
**9**	H	C_6_H_11_	28.32	73.58	>100	>100	>168
**10**	*i*-Pr	*i*-Pr	>100	84.56	98.14	>100	>180
**11**	Et	Et	66.02	>100	97.32	>100	>176
**12**	H	CHCH_3_C_2_H_5_	57.05	84.78	>100	>100	>175
**13**	H	*t*-Bu	>100	>100	>100	>100	>170
**14**	H	C_2_H_4_OH	87.72	66.01	89.01	>100	>180
**15**	C_2_H_4_OH	C_2_H_4_OH	35.14	42.94	37.85	>100	106.1–8
**16**	CH_2_CH_2_CH_2_CH_2_		>100	81.84	74.87	>100	116.5–7.4
ISL	-	-	36.90	44.11	58.43	>100	

^a^ PC-3, human prostate cancer cells; MCF-7, human breast cancer cells; HO-4980, human ovarian cancer cells.

For PC-3, compounds **9** and **15** had stronger growth inhibition effect, and the IC_50_ were 28.32 µM and 35.14 µM for 72 h, respectively. These two compounds showed stronger cytotoxicities than ISL. For HO-8910, **15** showed the strongest cytotoxicity with the IC_50_ values of 37.85 µM. For MCF-7, only **15** resulted in strong growth inhibition. Compound **15** was more potent than ISL against HO-8910 and MCF-7. Besides, the monoethanolamine substituted compound **14** showed much improved activity compared with the other derivatives, while compound **15** containing two hydroxyl attached with the nitrogen atom showed the most potent cytotoxicity. We conclude that amines containing hydroxyl located in 3′-position of the A ring could be highly beneficial for the activity of the compounds. In addition, compound **15** attached with amines containing two hydroxyl groups resulted in more potent cytotoxicities than compound **14** modified with amines containing only one hydroxy. Therefore, ISL derivative **15** was used to evaluate anti-tumor activity in S180 tumor-bearing mice for further studies.

**Table 2:** Effect of ISL and ISL derivative **15** (40 mg/kg, 80 mg/kg) on tumor weight and inhibitory ratio in S180 tumor-bearing mice.

Group	Treatment	Dose	Mice Number		TW (g) *x* ± SD	BWC ^a^ (%)	TIR ^b^ (%)
		(mg/kg)	Begin	End			
I	normal saline	-	10	10	1.23 ± 0.534 **	+4.4	-
II	ISL	40	10	10	0.85 ± 0.369 **	−3.0	32.44
III	ISL derivative **15**	40	10	10	0.74 ± 0.618 *	+12.8	39.72
IV	ISL derivative **15**	80	10	10	0.35 ± 0.442 **	+7.8	71.68

Student’s *t*-test was used to compare tumor volumes of treated mice. * *p* values < 0.05 were considered significant, ** *p* values < 0.01 were considered very significant. ^a^ Percentage of mice body-weight change (BWC) after drug treatment: BWC% = (mean BW final day/mean BW first day × 100) − 100; ‘‘+” means bodyweight increase; ‘‘−” means body-weight decrease. ^b^ Tumor inhibitory rate: TIR% = (1 − average tumor weight of experimental group/the average tumor weight of model control group).

**Figure 2:**
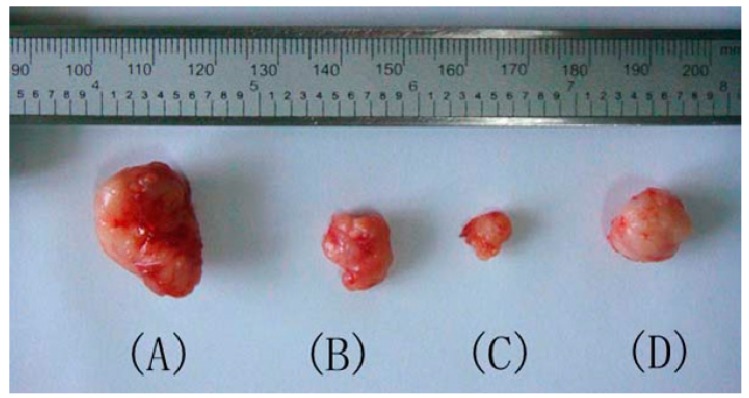
Effect of ISL and ISL derivative **15** on S180 tumor growth. ISL (40 mg/kg) was used as control drug. (**A**): Untreated negative control; (**B**): Treatment with low dose ISL derivative **15** (40 mg/kg); (**C**): Treatment with high dose ISL derivative **15** (80 mg/kg); (**D**): Treatment with ISL (40 mg/kg) as positive control.

#### 2.2.2. *In Vivo* Toxicity and Antitumor Studies

S180 tumor-bearing mice were treated with 80 or 40 mg/kg ISL derivative **15** and tumor inhibition rates of 71.68% and 39.72%, respectively, were obtained (Table 2). For comparison, ISL (40 mg/kg) was analyzed as positive control. Changes in body weight were used as parameter for toxicity. Loss of body weight was not found after exposure of animals to 80 or 40 mg/kg ISL derivative **15**, whereas ISL caused a slight weight loss in animals. Tumors were surgically removed after treatment with ISL and derivative **15**, and the results showed significant inhibition of tumor growth (Figure 2). The activity of ISL derivative **15** in S180 tumor-bearing mice indicated that ISL derivative **15** might be an attractive candidate for clinical use in oncology.

## 3. Experimental Section

### 3.1. Chemistry

^1^H (500 MHz) and ^13^C (125 MHz) NMR spectra were recorded with a Bruker instrument, and reported with TMS as internal standard and DMSO. Chemical shifts (δ values) and coupling constants (*J* values) are given in ppm and Hz. MS analysis was carried out on an API-3000 LC-MS-MS instrument. The melting points were determined on a WRS-1B digital melting point apparatus (Shanghai Precision & Scientific Instruments Co., Shanghai, China). Elemental analyses were carried out in a EA3000 CHNS-O analyzer. TLC analysis was carried out on silica gel plates GF254. Flash chromatography (Lisure Science (Suzhou) Co., Ltd., Suzhou, China) was performed with silica gel 300–400 mesh using a glass column. Unless otherwise indicated, reagents and solvents were obtained from commercial suppliers. 

Procedure for chalcone **6**: *p*-hydroxybenzaldehyde (219.8 mg, 1.8 mmol) and pyridine-PPTS (45.2 mg, 0.18 mmol) were dissolved in dichloromethane (5 mL),2,3-dihydropyran (0.25 mL, 2.7 mmol) was dropwise added. The reaction was stirred at room temperature for 4 h, the mixture was treated with saturated NaCl and extracted with ether. Solvent was removed under reduced pressure to obtain oily crude product (Yield: 94.4%). Similarly, 2,4-dihydroxyacetophenone (152.2 mg, 1.0 mmol), pyridine-PPTS (25.1 mg, 0.1 mmol), and 2,3-dihydropyran (0.14 mL, 1.5 mmol) were added into a round bottom flask and stirred for 2 h, NaHCO_3_ (80.0 mg, 1 mmol) was added, continued for another 2.5 h, the mixture was filtered. Oily crude **4** product was obtained after solvent was removed under reduced pressure (Yield: 91.2%). These two hydroxyl-proctected products were dissolved in CH_3_OH (5 mL), Ba(OH)_2_ (428.4 mg, 2.5 mmol) was added as catalyst. The reaction was stirred at 45 °C (TLC detected) and after 12 h it was diluted with CH_2_Cl_2_. Then, HCl was added to adjust the system to weak acid. The mixture was extracted with CH_2_Cl_2_. The condensation product **5** was obtained after solvent was removed *in vacuo* and was following dissolved in CH_3_OH (Yield: 77.9%). PTSA (4.8 mg, 0.028 mmol) was added and the mixture was stirred at room temperature for 6 h, the reaction mixture was poured into ice water and yellow crystals were filtered, washed and then dried. Finally, target compound **6** was obtained (Yield: 95.8%).

General procedure for ISL derivatives **7**–**16**: ISL 128 mg (0.5 mmol) were put into a round bottom flask, and substituted amines (0.6 mmol) were added. Temperature was kept at 16–18 °C. The mixture of 36% formaldehyde solution (0.046 ml, 0.6 mmol) and acetic acid (2 ml) was quickly and dropwise added under electromagnetic stirring. The reaction immediately stopped, when the agents has been dropwise added. The mixture was treated with water and extracted with EtOAc. The organic phase was dried over Na_2_SO_4_. The solvent was removed under reduced pressure, and the crude mixture was purified by column chromatography with EtOAc/MeOH (5:1 to 1:2 *v*/*v*) as eluent to afford the target compounds. 

*(E)-1-(3-(dimethylaminomethyl)-2,4-dihydroxyphenyl)-3-(4-hydroxyphenyl)prop-2-en-1-one* (**7**). According to the general procedure for ISL derivatives using dimethylamine as amine, the lead compound was obtained as yellow powder. Weight: 43.5 mg. Yield: 27.8%. Melting point: >176 °C; ^1^H-NMR (500 MHz, DMSO): 2.41 (6H, s, (CH_3_)_2_N-), 3.84 (2H, s, -CH_2_N), 6.22 (1H, d, *J* = 9 Hz, H3), 6.82~6.84 (2H, m, H5, H6), 7.67~7.75 (4H, m, H2′,3′, 5′, 6′), 8.03 (1H, d, *J* = 9 Hz, CH_2_); ^13^C-NMR (125 MHz, DMSO): 190.65, 172.59, 170.20, 164.47, 160.58, 143.63, 132.05, 131.46, 126.36, 118.04, 116.29, 111.01, 110.15, 106.89, 54.17, 43.76. ESI-MS, *m*/*z* [M+H]^+^ 314.3. For C_18_H_19_NO_4_: C, 68.99; H, 6.11; N, 4.47. Found: C, 68.90; H, 6.08; N, 4.42.

*(E)-1-(2,4-dihydroxy-3-(morpholinomethyl)phenyl)-3-(4-hydroxyphenyl)prop-2-en-1-one* (**8**). According to the general procedure for ISL derivatives using morpholine as amine, the lead compound was obtained as yellow powder. Weight: 62.84 mg. Yield: 35.4%. Melting point: >180 °C; ^1^H-NMR (500 MHz, DMSO): 2.51 (4H, s, -(CH2)_2_N), 3.61 (4H, s, -(CH_2_)_2_O), 3.74 (2H, s, -CH_2_N), 6.38 (1H, d, *J* = 9 Hz, H3), 6.83~6.85 (2H, m, H5, H6), 7.75~7.77 (4H, m, H2′, 3′, 5′, 6′), 8.13 (1H, d, *J* = 9 Hz, H2); ^13^C-NMR (125 MHz, DMSO): 191.68, 165.27, 163.75, 160.27, 44.30, 131.47, 131.24, 125.75, 117.31, 115.82, 112.35, 107.99, 107.75, 66.00, 52.54, 51.99. ESI-MS, *m*/*z* [M+H]^+^ 356.3. Anal. Calcd. For C_20_H_21_NO_5_: C, 67.59; H, 5.96; N, 3.94. Found: C, 67.54; H, 5.89; N, 3.87.

*(E)-1-(3-(cyclohexylaminomethyl)-2,4-dihydroxyphenyl)-3-(4-hydroxyphenyl)prop-2-en-1-one* (**9**). According to the general procedure for ISL derivatives using cyclohexylamine as amine, the lead compound was obtained as yellow powder. Weight: 60.9 mg. Yield: 33.2%. Melting point: >168 °C; ^1^H-NMR (500 MHz, DMSO): 1.14~1.99 (10H, m, -CH_2_), 2.74 (1H, s, -CHN), 4.01 (2H, s, -CH_2_N), 5.99 (1H, d, *J* = 9 Hz, H3), 6.80~6.82 (2H, m, H5, H6), 7.59~7.70 (4H, m, H2′, 3′, 5′, 6′), 7.88 (1H, d, *J* = 9 Hz, H2); ^13^C-NMR (125 MHz, DMSO): 188.20, 175.04, 164.28, 159.67, 141.63, 131.33, 130.63, 126.14, 117.91, 115.74, 111.99, 108.27, 105.14, 54.78, 29.88, 25.02, 23.93. ESI-MS, *m*/*z* [M+H]^+^ 368.4. Anal. Calcd. For C_22_H_25_NO_4_: C, 71.91; H, 6.86; N, 3.81. Found: C, 71.89; H, 6.87; N, 3.83.

*(E)-1-(3-(diisopropylamino)-methyl)-2,4-dihydroxyphenyl)-3-(4-hydroxyphenyl)prop-2-en-1-one* (**10**). According to the general procedure for ISL derivatives using diisopropylamine as amine, the lead compound was obtained as yellow powder. Weight: 57.56 mg. Yield: 31.2%. Melting point: >180 °C; ^1^H-NMR (500 MHz, DMSO): 1.12 (12H, s, -CH_3_), 3.23 (2h, s, -CHN), 3.96 (2H, s, -CH_2_N), 6.16 (1H, d, *J* = 9 Hz, H3), 6.82~6.84 (2H, m, H5, H6), 7.70~7.74 (4H, m, H2′, 3′, 5′, 6′), 8.00 (1H, d, *J* = 9 Hz, H2); ^13^C-NMR (125 MHz, DMSO): 190.52, 170.27, 163.17, 160.02, 143.27, 131.01, 125.89, 117.50, 115.78, 110.58, 109.59, 106.60, 49.11, 30.66, 18.27. ESI-MS, *m*/*z* [M+H]^+^ 370.3. Anal. Calcd. For C_22_H_27_NO_4_: C, 71.52; H, 7.37; N, 3.79. Found: C, 71.51; H, 7.35; N, 3.75.

*(E)-1-(3-((diethylamino)methyl)-2,4-dihydroxyphenyl)-3-(4-hydroxyphenyl)prop-2-en-1-one* (**11**). According to the general procedure for ISL derivatives using diaethylamin as amine, the lead compound was obtained as yellow powder. Weight: 72.97 mg. Yield: 42.8%. Melting point: >176 °C; ^1^H-NMR (500 MHz, DMSO): 1.09 (6H, t, *J* = 7 Hz, CH_3_), 2.71~2.75 (4H, q, *J* = 7 Hz, (CH_2_)_2_N-), 3.92 (2H, s, -CH_2_N), 6.19 (1H, d, *J* = 9 Hz, H3), 6.82~6.84 (2H, m, H5, H6), 7.67~7.75 (4H, m, H2′, 3′, 5′, 6′), 8.02 (1H, d, *J* = 9 Hz, H2); ^13^C-NMR (125 MHz, DMSO): 190.80, 170.48, 164.19, 160.52, 143.69, 131.85, 131.48, 126.38, 118.03, 116.27, 111.08, 110.13, 106.68, 49.28, 46.45, 10.79. ESI-MS, *m*/*z* [M+H]^+^ 342.1. Anal. Calcd. For C_20_H_23_NO_4_: C, 70.36; H, 6.79; N, 4.10. Found: C, 70.43; H, 6.74; N, 4.20.

*(E)-1-(3-((sec-butylamino)methyl)-2,4-dihydroxyphenyl)-3-(4-hydroxyphenyl)prop-2-en-1-one* (**12**). According to the general procedure for ISL derivatives using sec-butylamine as amine, the compound was obtained as yellow powder. Weight: 46.71 mg. Yield: 27.4%. Melting point: >175 °C; ^1^H-NMR (500 MHz, DMSO): 0,92~0.93 (6H, d, *J* = 7 Hz, CH3), 1.82~1.88 (1H, m, -CHN), 2.56 (2H, d, *J* = 7 Hz, -CH_2_-), 3.99 (2H, s, -CH_2_N), 6.04 (1H, d, *J* = 9 Hz, H3), 6.81~6.83 (2H, m, H5, H6), 7.61~7.71 (4H, m, H2′, 3′, 5′, 6′), 7.91 (1H, d, *J* = 9 Hz, H2); ^13^C-NMR (125 MHz, DMSO): 189.17, 174.56, 164.68, 160.22, 142.45, 131.84, 131.19, 126.59, 118.34, 116.24, 111.95, 109.23, 105.78, 54.73, 44.17, 26.79, 20.58. ESI-MS, *m*/*z* [M+H]^+^ 342.3. Anal. Calcd. For C_20_H_23_NO_4_: C, 70.36; H, 6.79; N, 4.10. Found: C, 70.33; H, 6.84; N, 4.07.

*(E)-1-(3-((tert-butylamino)methyl)-2,4-dihydroxyphenyl)-3-(4-hydroxyphenyl)prop-2-en-1-one* (**13**). According to the general procedure for ISL derivatives using tert-butylamine as amine, the lead compound was obtained as yellow powder. Weight: 65.64 mg. Yield: 38.5%. Melting point: >170 °C; ^1^H-NMR (500 MHz, DMSO): 1.27 (9H, s, CH3), 3.98 (2H, s, -CH_2_N), 5.99 (1H, d, *J* = 9 Hz, H3), 6.81~6.83 (2H, m, H5, H6), 7.59~7.70 (4H, m, H2′, 3′, 5′, 6′), 7.88 (1H, d, *J* = 9 Hz, H2); ^13^C-NMR (125 MHz, DMSO): 188.59, 164.80, 160.18, 142.09, 131.89, 126.63, 118.39, 116.24, 112.66, 108.63, 105.64, 54.03, 37.54, 26.47. ESI-MS, *m*/*z* [M+H]^+^ 342.2. Anal. Calcd. For C_20_H_23_NO_4_: C, 70.36; H, 6.79; N, 4.10. 

*1-(2,4-dihydroxy-3-((2-hydroxyethylamino)methyl)phenyl)-3-(4-hydroxyphenyl)propen-1-one* (**14**). According to the general procedure for ISL derivatives using monoethanolamine as amine, the lead compound was obtained as yellow powder. Weight: 41.62 mg. Yield: 25.3%. Melting point: >180 °C; ^1^H-NMR (500 MHz, DMSO): 2.82 (2H, s, -CH_2_OH), 3.58 (2H, s, -CH_2_N), 4.01 (2H, s, -CH_2_N), 6.03 (1H, d, *J* = 8 Hz, H3), 6.82~6.83 (2H, m, H5, H6), 7.63~7.69 (4H, m, H2′, 3′, 5′, 6′), 7.90 (1H, d, *J* = 8 Hz, H2); ^13^C-NMR (125 MHz, DMSO): 165.03, 160.16, 142.20, 131.89, 131.14, 126.64, 118.40, 116.23, 112.23, 108.95, 105.79, 58.14, 49.26, 43.30. ESI-MS, *m*/*z* [M+H]^+^ 330.5. Anal. Calcd. For C_18_H_19_NO_5_: C, 65.64; H, 5.81; N, 4.25. Found: C, 65.66; H, 5.85; N, 4.21.

*(E)-1-(3-((bis(2-hydroxyethyl)amino)methyl)-2,4-dihydroxyphenyl)-3-(4-hydroxyphenyl)prop-2-en-1-one* (**15**). According to the general procedure for ISL derivatives using diethanolamine as amine, the lead compound was obtained as yellow powder. Weight: 69.94 mg. Yield: 37.5%. Melting point: 106.1–106.8 °C; ^1^H-NMR (500 MHz, DMSO): 2.74 (4H, s, -(CH_2_)_2_N), 3.58~3.60 (4H, t, *J* = 6 Hz, -CH_2_OH), 3.98 (2H, s, -CH2N), 6.28 (1H, d, *J* = 9 Hz, H3), 6.83~6.85 (2H, m, H5, H6), 7.74~7.76 (4H, m, H2′, 3′, 5′, 6′), 8.08 (1H, d, *J* = 9 Hz, H2); ^13^C-NMR (125 MHz, DMSO): 191.61, 172.47, 168.05, 164.05, 160.66, 144.30, 131.88, 131.6, 212.63, 1117.93, 116.30, 112.05, 109.29, 108.12, 58.07, 55.86, 50.29, 21.53. ESI-MS, *m*/*z* [M+H]^+^ 374.1. Anal. Calcd. For C_20_H_23_NO_6_: C, 64.33; H, 6.21; N, 3.75. Found: C, 64.32; H, 6.27; N, 3.69.

*(E)-1-(2,4-dihydroxy-3-(pyrrolidin-1-ylmethyl)phenyl-3-(4-hydroxyphenyl)prop-2-en-1-one* (**16**). According to the general procedure for ISL derivatives using pyrrolidine as amine, the lead compound was obtained as yellow powder. Weight: 60.0 mg. Yield: 35.4%. Melting point: 116.5–117.1 °C; ^1^H-NMR (500 MHz, DMSO): 1.84 (4H, s, -CH2-CH2-), 2.82~2.84 (4H, t, *J* = 6 Hz, (CH_2_)_2_N-), 3.99 (2H, s, -CH_2_N), 6.18 (1H, d, *J* = 9 Hz, H3), 6.82~6.84 (2H, m, H5, H6), 7.65~7.73 (4H, m, H2′, 3′, 5′, 6′), 8.00 (1H, d, *J* = 9 Hz, H2); ^13^C-NMR (125 MHz, DMSO): 190.20, 172.54, 171.03, 164.57, 160.48, 143.31, 132.02, 131.39, 126.43, 118.10, 116.28, 110.64, 110.51, 107.105, 334 , 50.31, 23.54. ESI-MS, *m*/*z* [M+H]^+^ 340.3. Anal. Calcd. For C_20_H_21_NO_4_: C, 70.78; H, 6.24; N, 4.13. Found: C, 70.85; H, 6.20; N, 4.15.

### 3.2. Biological Assays

#### 3.2.1. Cytotoxicity Assay

Cytotoxicity was evaluated against four human cell lines (MCF-7 breast cancer, PC-3 prostatic cancer, HO-8910 ovarian cancer, and THP1 normal cells). Briefly, MCF-7, PC-3 and HO-8910 cells were separately plated in 96-well culture plates (1 × 10^5^ cells/well). After 24 h incubation, cells were treated with compounds **7**–**16** (0, 3.13, 6.25, 12.5, 25, 50 and 100 µM) for 72 h. MTT solution (5 mg/mL) was then added to each well. After 4 h incubation, the formazan precipitate was dissolved in 100 mL dimethyl sulfoxide (DMSO), and then the absorbance was measured in an ELISA reader (Thermo Molecular Devices Co., Union City, CA, USA) at 570 nm. The cell viability ratio was calculated by the following formula: Inhibitory ratio (%) = (1 − OD_treated_/OD_control_) × 100%.

#### 3.2.2. Animal Treatment

Female BALB/c mice (6–8 weeks old, 25 ± 2 g) were obtained from the Third Affiliated Hospital of Harbin Medical University. S180 sarcoma cells were purchased from the Experimental Animal Center of Peking University Health Science Center (Heilongjiang, China). The animals were housed in polypropylene cages and maintained under controlled conditions of 12 h light/12 h dark photoperiod and 55 ± 5% relative humidity at room temperature (28 ± 2 °C). After fed in our facility for 1 week, 40 mice were induced S180 tumor cells according to Wu *et al.* with some modification [32]. All mice were subcutaneously implanted with 1 × 10^6^ cells/mice on the right flank. After inoculation for 24 h, all animals were randomly divided into one untreated control group, one treated positive control group and two treated experimental groups (each group contained 10 mice).

Group I (untreated control) received orally the same volume of 0.9% normal saline once per day as the treated groups. Group II (treated positive control) received orally ISL at a dosage of 40 mg/kg body weight once per day. Group III (low dose of ISL derivative **15** treatment) received ISL derivative **15** orally (dissolved in 0.2 mL normal saline) at a dosage of 40 mg/kg body weight once per day. Group IV (high-dose of ISL derivative **15** treatment) received ISL derivative **15** orally (dissolved in 0.2 mL normal saline) at a dosage of 80 mg/kg body weight once per day.

## 4. Conclusions

A series of novel aminomethylated derivatives of ISL were synthesized and their anti-tumor activities *in vitro* were evaluated against three cancer cell lines. Through the results, we found that ISL derivatives contained two or more aminemethyl and nitrogen connected with methylene substituent exhibited considerable cytotoxicity. S180 tumor-bearing mice were treated with 80 or 40 mg/kg ISL derivative **15** and tumor inhibition rates were 71.68% and 39.72%, respectively. Our results indicated that ISL derivative **15** might be an attractive candidate for clinical use in oncology in the future and the scheme we designed to synthesize ISL derivatives might become a useful method for chemical synthesis and modification of flavone drugs.

## Supplementary Materials

Supplementary materials can be accessed at: http://www.mdpi.com/1420-3049/19/11/17715/s1.

## Supplementary Files

Supplementary File 1
