# Supplementary material for: Synthesis and Anti-Tumor Activity of Novel Aminomethylated Derivatives of Isoliquiritigenin
Source: Molecules. 2014 Oct 31;19(11):17715–26. doi: 10.3390/molecules191117715 (PMC6271340; doi:10.3390/molecules191117715)

Supplementary Materials

Figure S1. ^{1}H-NMR of compound **7**.

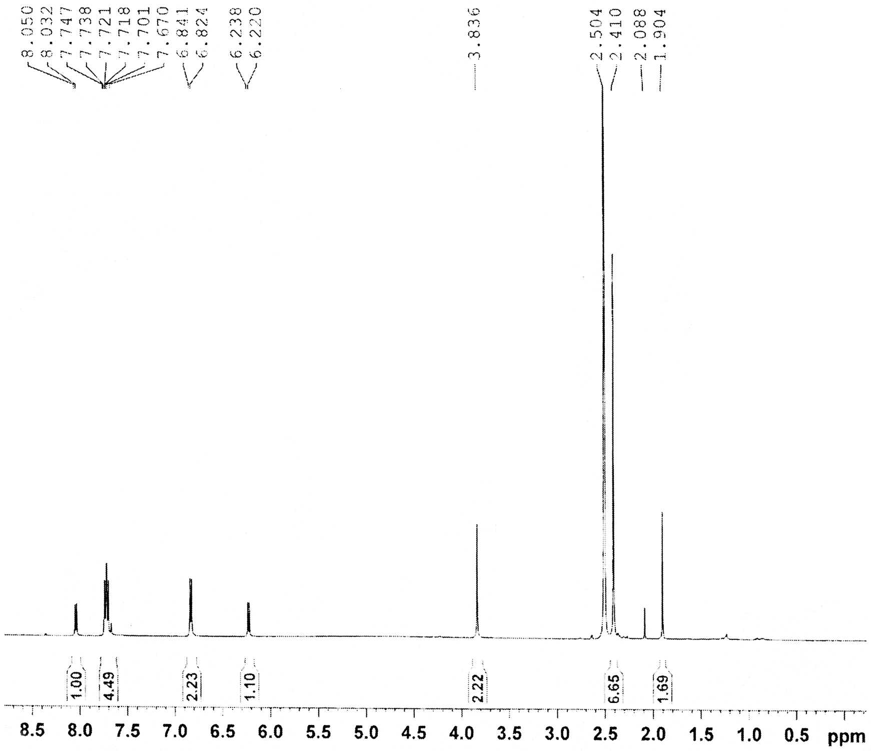

Figure S2. ^{13}C-NMR of compound **7**.

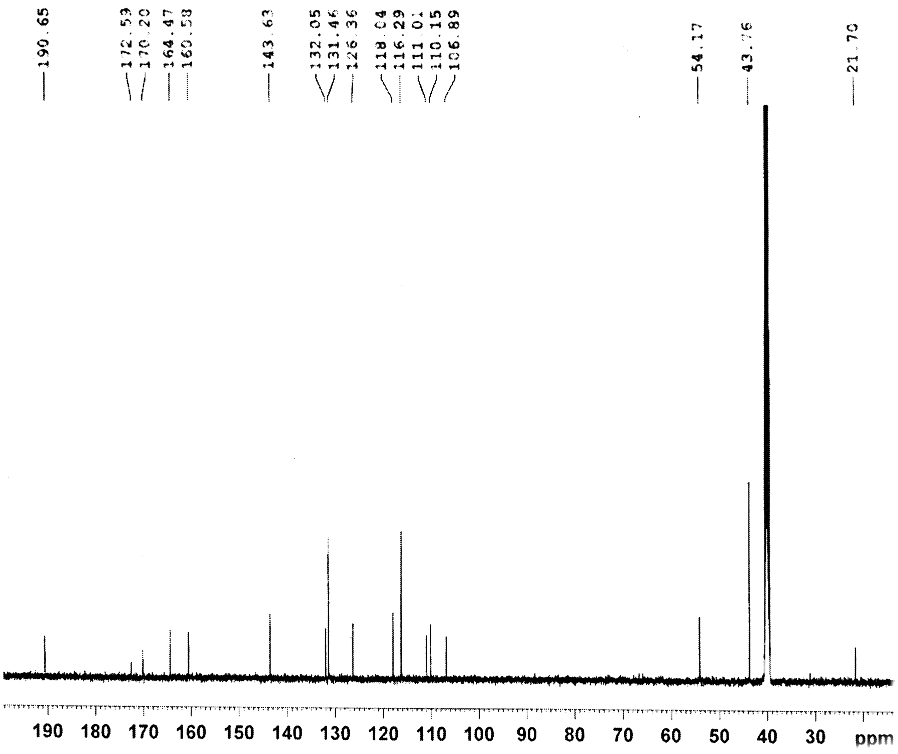

Figure S3. ^{1}H-NMR of compound **8**.

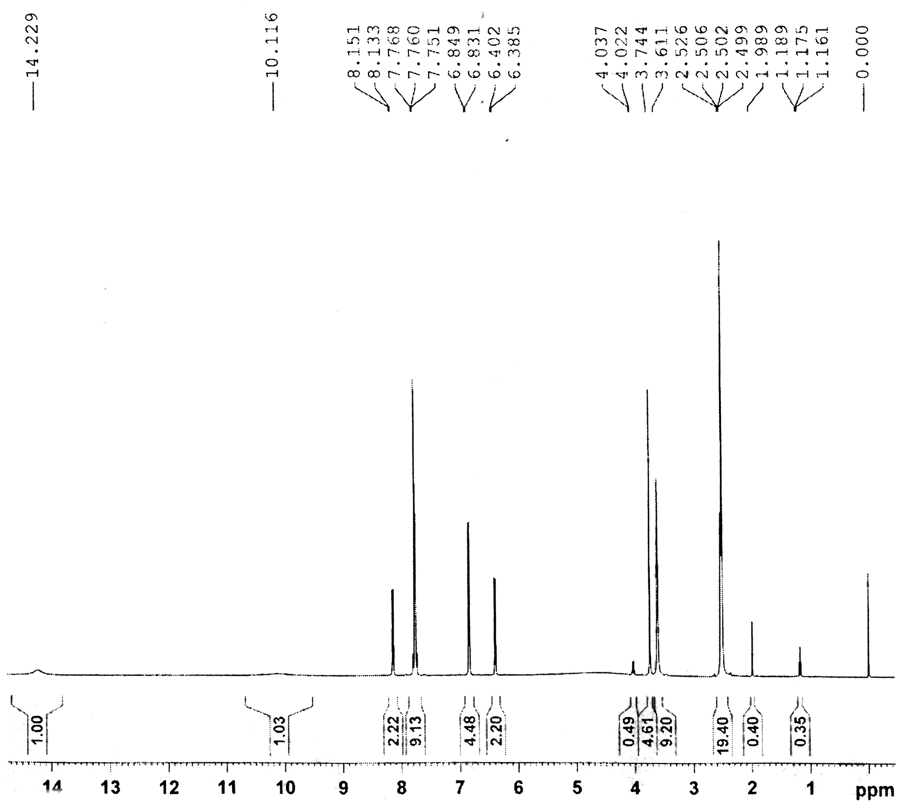

Figure S4. ^{13}C-NMR of compound **8**.

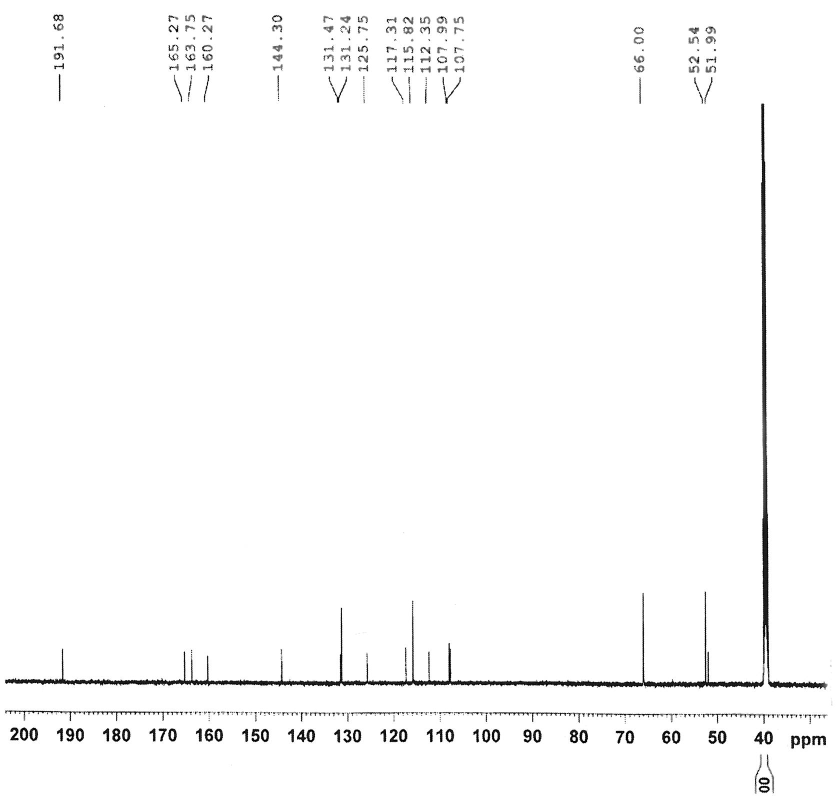

Figure S5. ^{1}H-NMR of compound **9**.

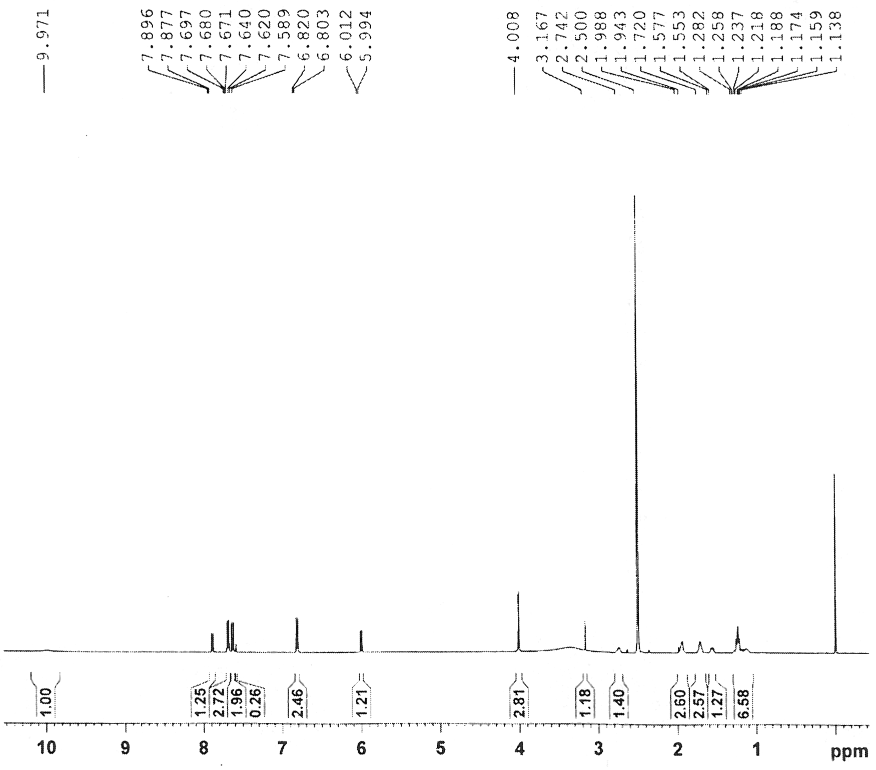

Figure S6. ^{13}C-NMR of compound **9**.

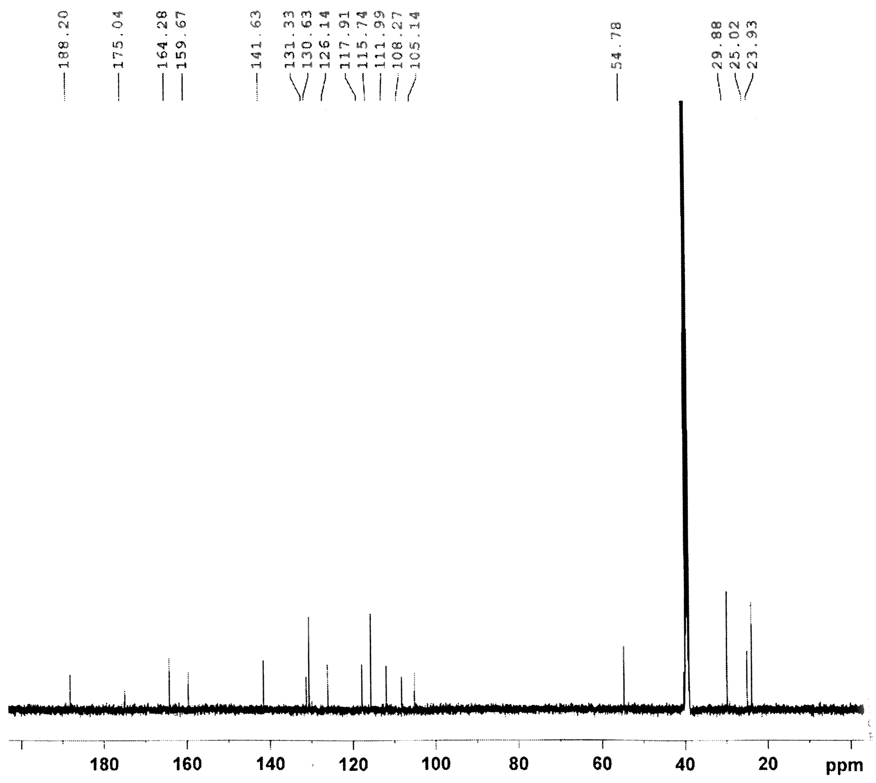

Figure S7. ^{1}H-NMR of compound **10**.

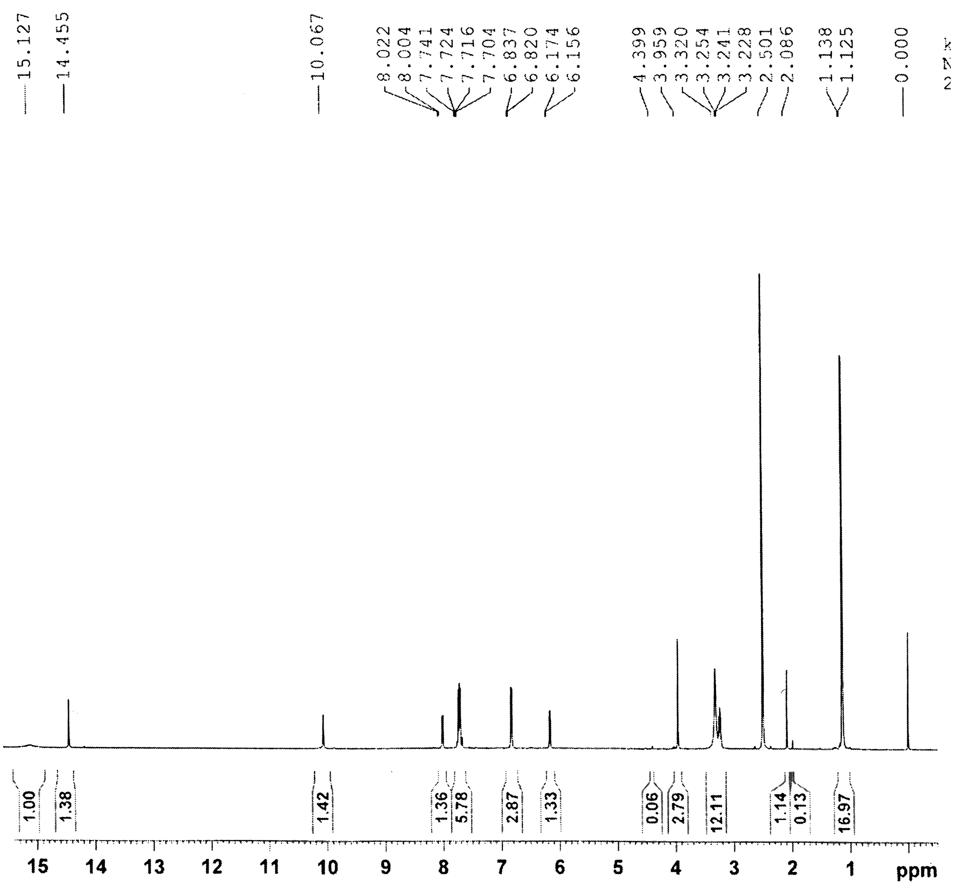

Figure S8. ^{13}C-NMR of compound **10**.

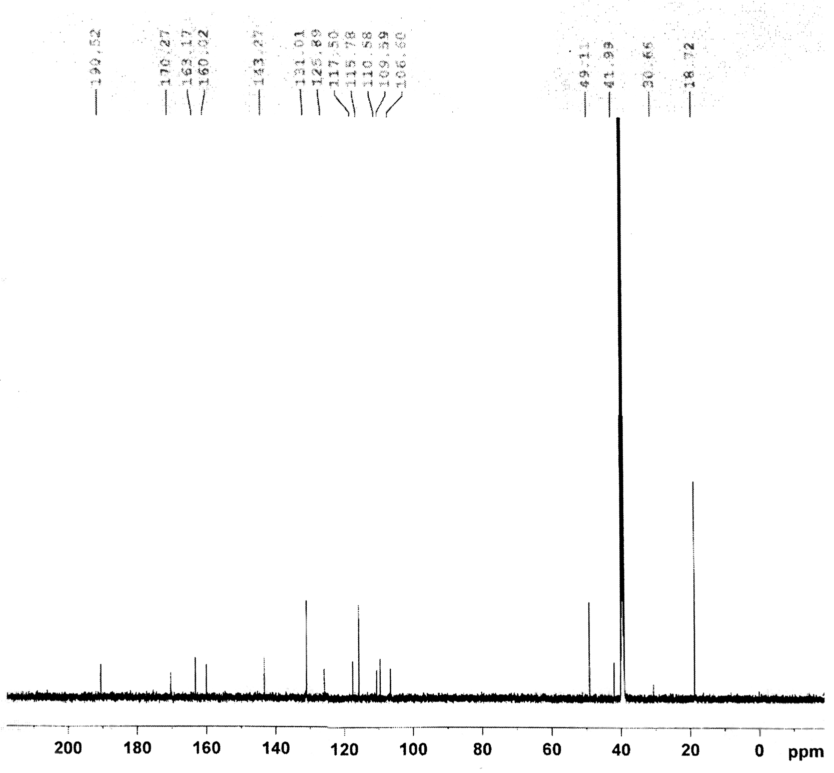

Figure S9. ^{1}H-NMR of compound **11**.

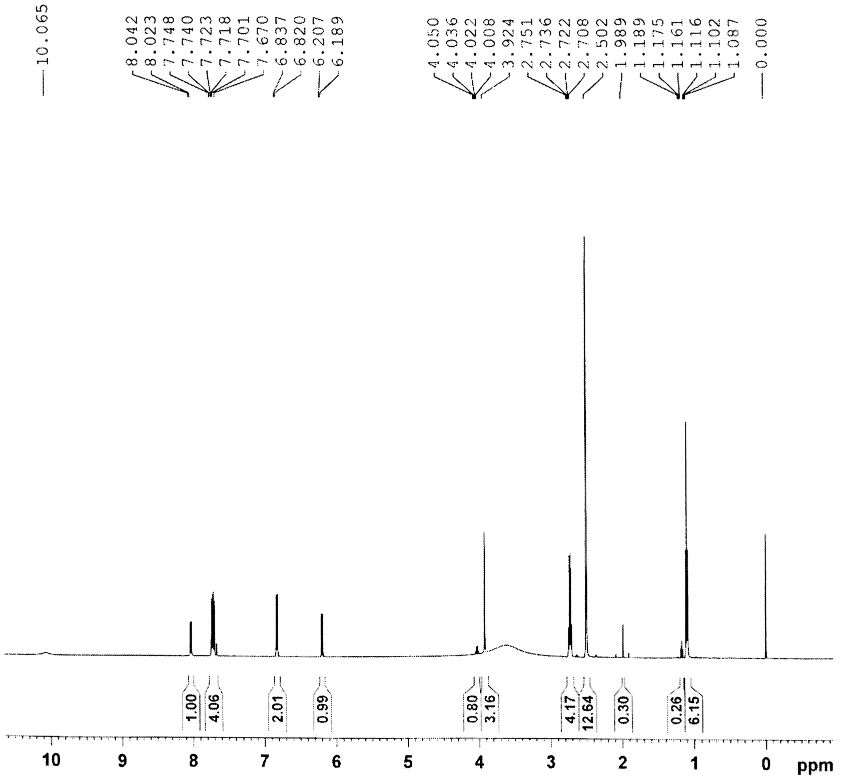

Figure S10. ^{13}C-NMR of compound **11**.

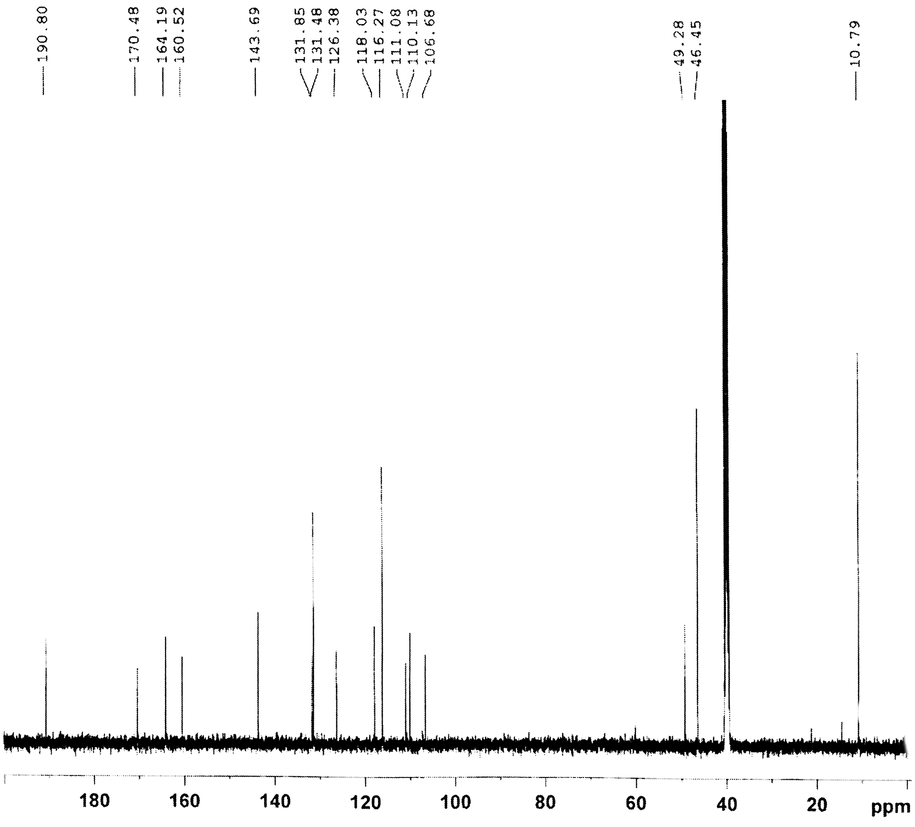

Figure S11. ^{1}H-NMR of compound **12**.

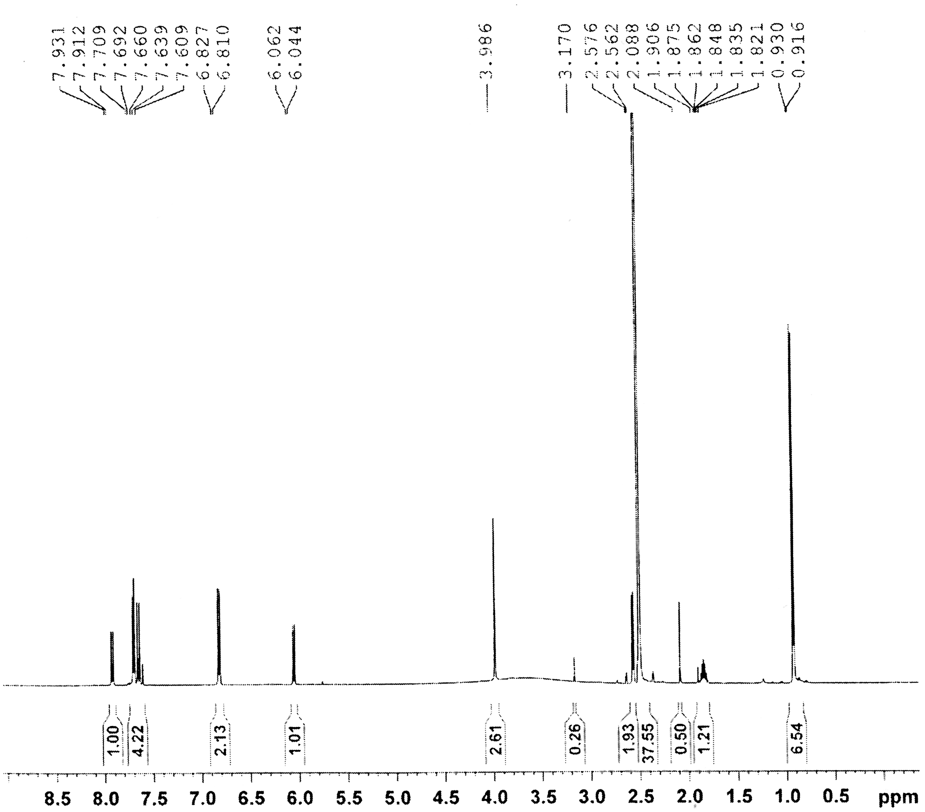

Figure S12. ^{13}C-NMR of compound **12**.

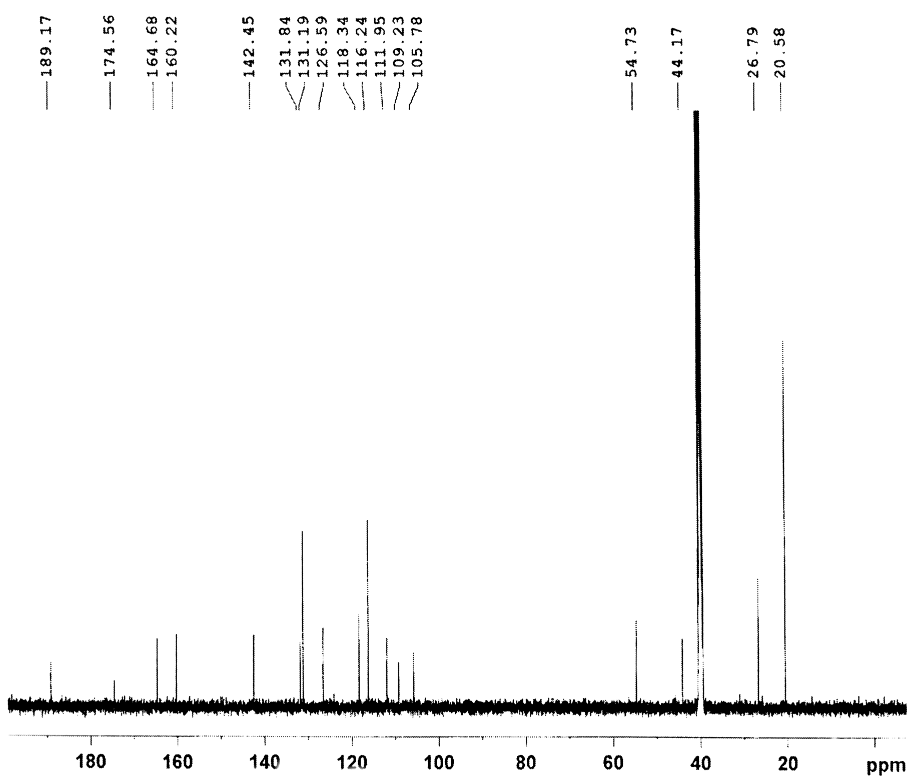

Figure S13. ^{1}H-NMR of compound **13**.

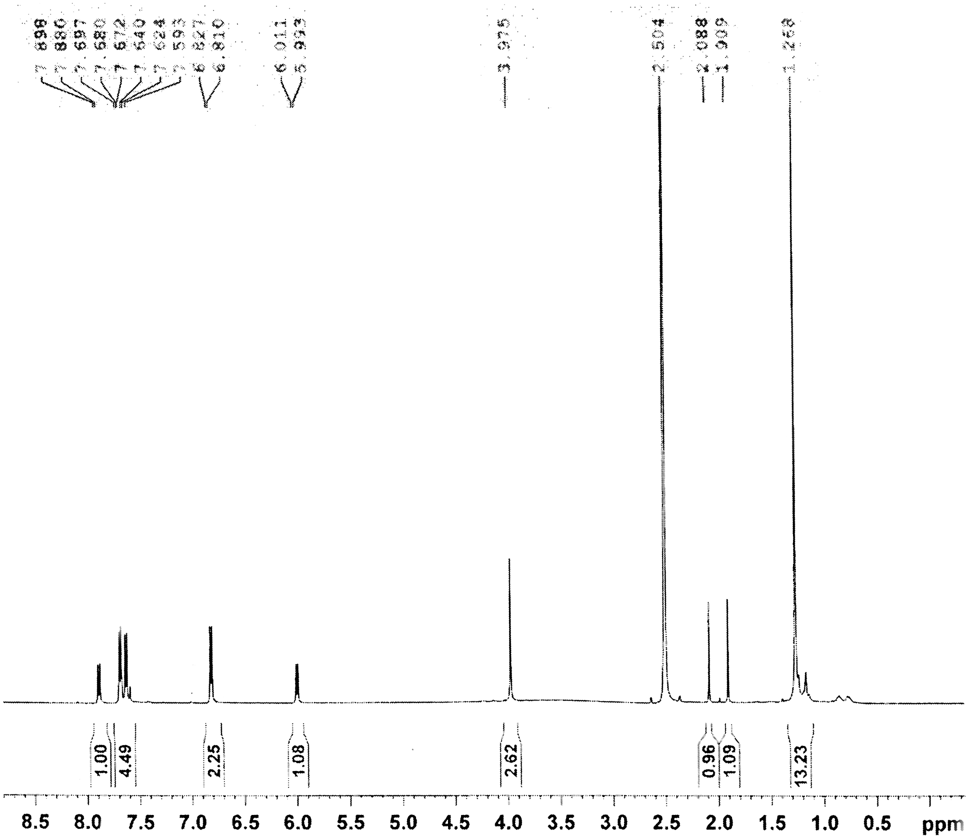

Figure S14. ^{13}C-NMR of compound **13**.

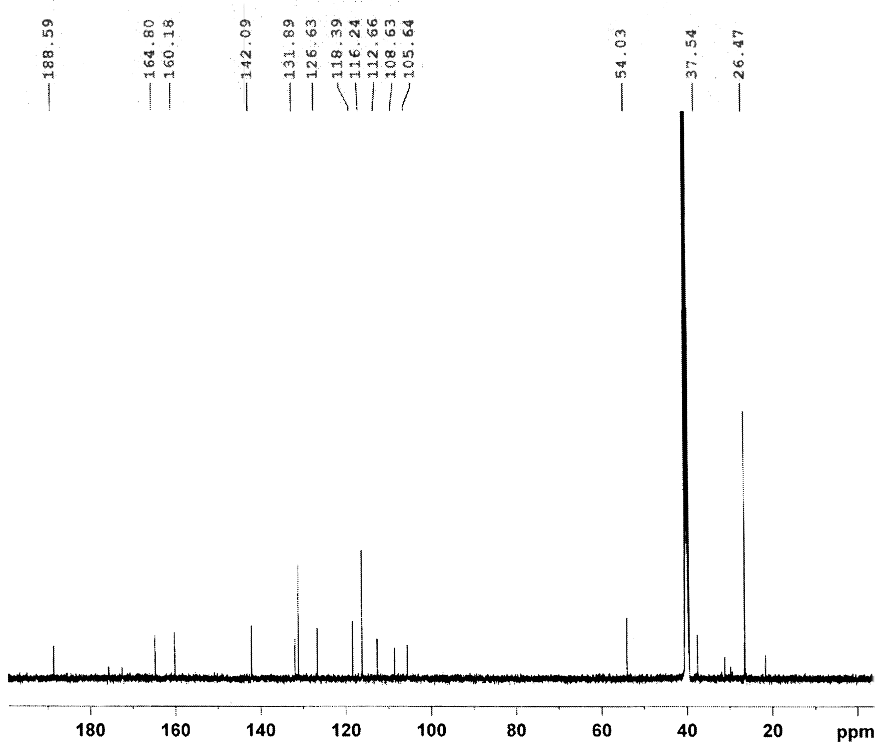

Figure S15. ^{1}H-NMR of compound **14**.

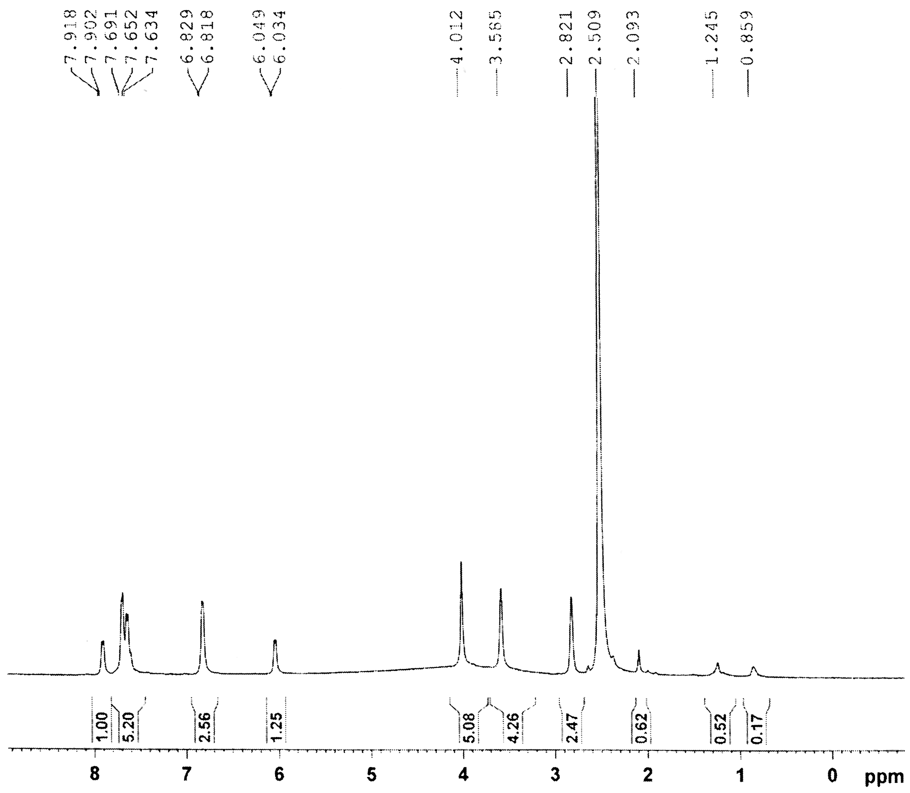

Figure S16. ^{13}C-NMR of compound **14**.

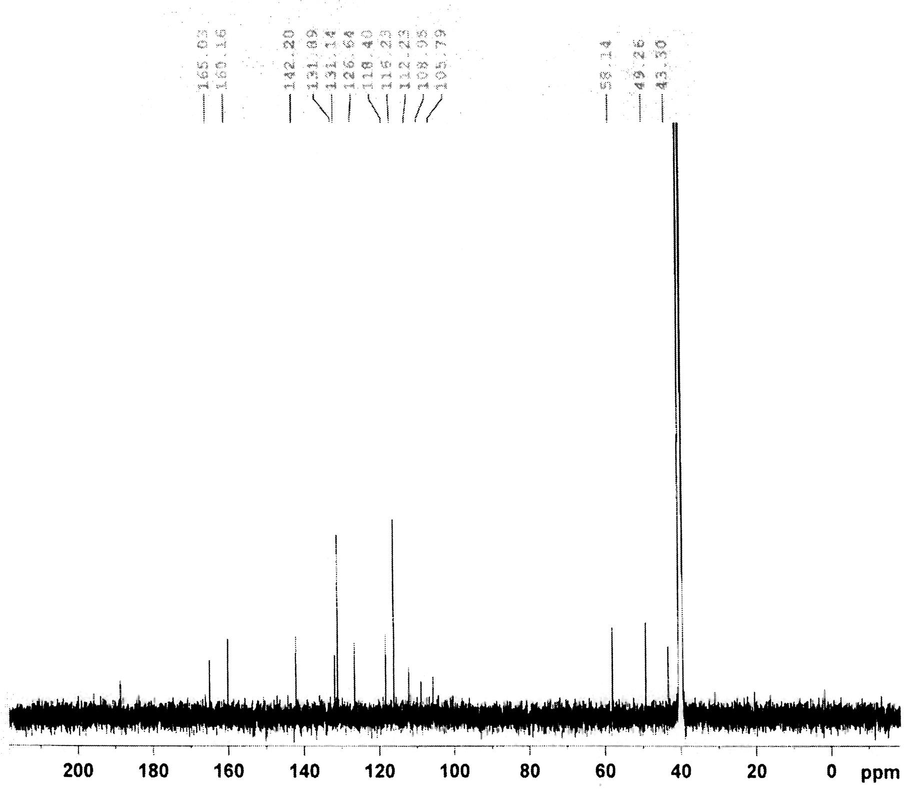

Figure S17. ^{1}H-NMR of compound **15**.

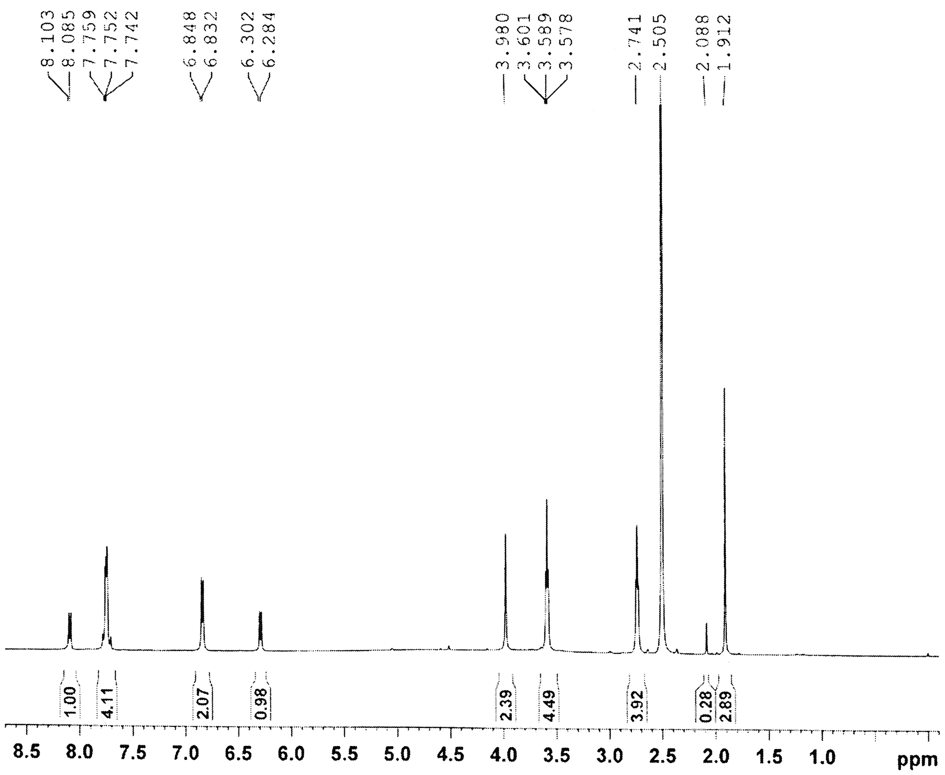

Figure S18. ^{13}C-NMR of compound **15**.

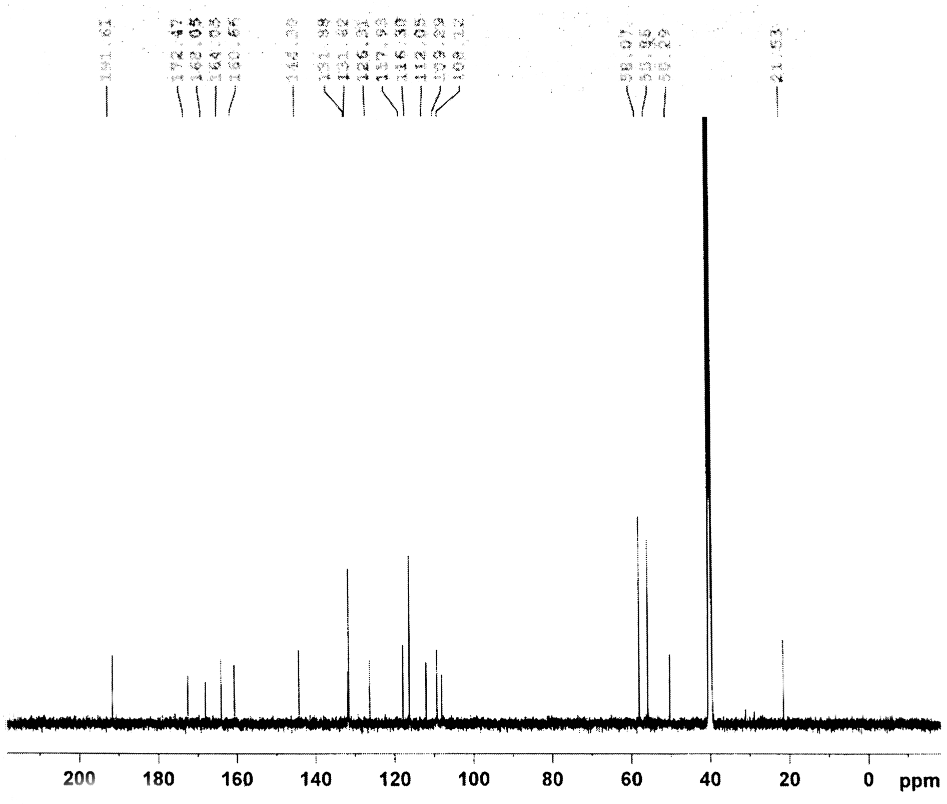

Figure S19. ^{1}H-NMR of compound **16**.

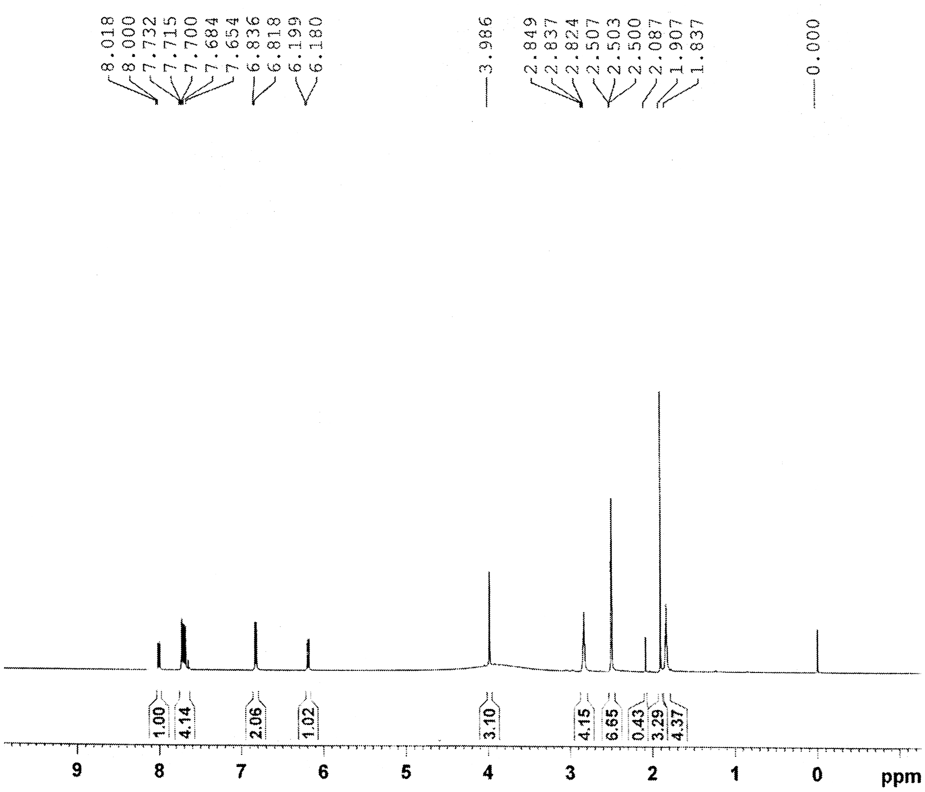

Figure S20. ^{13}C-NMR of compound **16**.

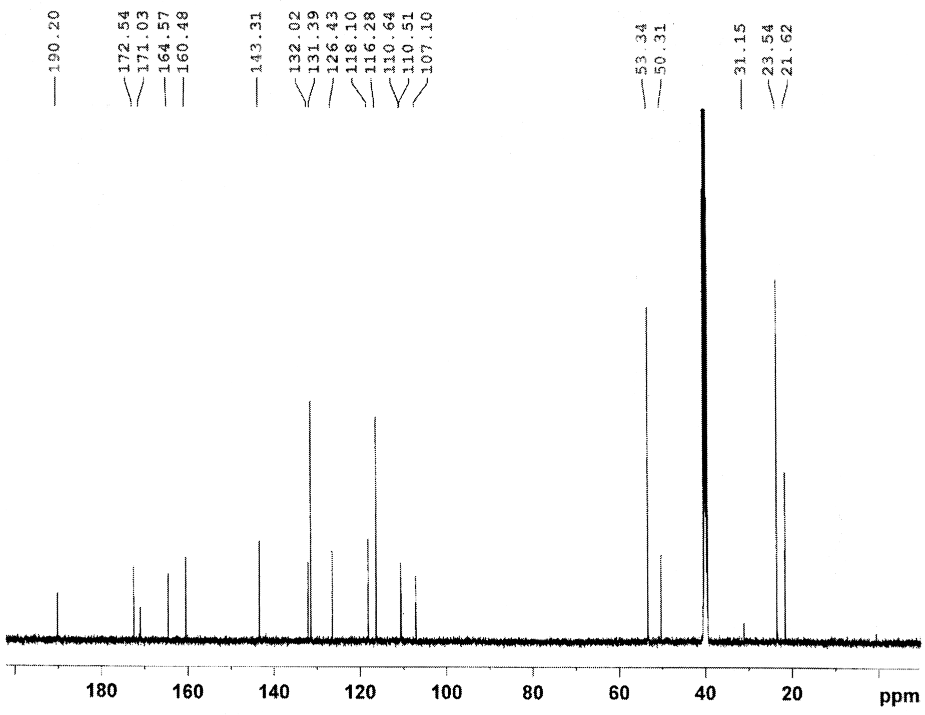

Supplement: Supplementary File 1 [file molecules-19-17715-s001.pdf]
